# Systematic Analysis of the Clinical Relevance of Cell Division Cycle Associated Family in Endometrial Carcinoma

**DOI:** 10.7150/jca.46324

**Published:** 2020-07-20

**Authors:** Wenchao Zhang, Xiaofeng Qiu, Di Sun, Danye Zhang, Yue Qi, Xiao Li, Bingying Liu, Juanjuan Liu, Bei Lin

**Affiliations:** 1 Affiliation: Department of Obstetrics and Gynecology, Shengjing Hospital of China Medical University, Liaoning, China, Post code: 110000.; 2 Affiliation: Key Laboratory of Maternal-Fetal Medicine of Liaoning Province, Key Laboratory of Obstetrics and Gynecology of Higher Education of Liaoning Province, Liaoning, China, Post code: 110000.; 3 Affiliation: Department of Obstetrics and Gynecology, Zaozhuang Shizhong District Maternal and Child Health Hospital, Shandong, China.

**Keywords:** CDCA, TCGA, endometrial carcinoma, prognosis

## Abstract

**Background**: Endometrial carcinoma (EC) is the most common cancer of female reproductive system, thus requiring for new effective biomarkers which could predict the onset of EC and worse prognosis. Cell Division Cycle Associated (CDCA) family plays indispensable roles in cell cycle process. However, no study has been focused on the role of CDCAs in EC. Our study aims to investigate the clinical relevance, potential biologic functions and molecular mechanisms of CDCAs in EC.

**Methods**: GEPIA, cBioPortal, GeneMANIA, Networkanalyst, TCGA-UCEC cohort were utilized in this study. **Results**: NUF2 and CDCA2/3/4/5/7/8 were significantly highly expressed in EC compared with normal tissues. The patients with high NUF2 and CDCA2/3/4/5/8 expression tended to develop to advanced FIGO stages, poor differentiation and worse prognosis(in both OS and RFS analyses) than those with low expression. By contrast, elevated CDCA7 was significantly associated with better prognosis. CBX2 exerted no significant prognostic impact on EC patients. Distinct patterns of the genetic alterations of CDCAs were observed in various histological subtypes of EC. The biological functions of NUF2 and CDCA2/3/4/5/8 were mainly related with the activation of the following pathway: cell cycle, DNA replication, base excision repair, mismatch repair, nucleotide excision repair, cellular senescence and p53 signaling pathway.

**Conclusions**: Our study provides new insight into the onset and progression of EC and proposes NUF2 and CDCA2/3/4/5/8 could act as oncogenes and have shown great diagnostic and prognostic promise in improving EC patient detection and survival prediction with accuracy.

## Introduction

Endometrial carcinoma (EC) is the most common cancer of female reproductive organs in the United States 1 and the second most common cancer worldwide, next to cervical cancer 2. It is estimated that 65,620 more cases will be diagnosed and 12,590 of them will die of it in the U.S. alone in 2020 1. Women who have problems like obesity 3, diabetes, high blood pressure, receiving estrogen/tamoxifen therapy or genetic Lynch syndrome are at higher risk of contracting EC 4.

EC is classified into two subtypes, based on clinical features and pathogenesis. Type I EC is endometroid, estrogen-dependent and represents 75-90% of EC 5, while type II endometrial cancer is non-endometroid (such as serous and clear-cell carcinoma), estrogen-independent and harbors mutant gene (p53, p16, etc.), which is usually associated with a higher risk of metastasis and worse survival outcome 6.

Abnormal uterine bleeding is the most frequent symptom of EC, but many other disorders give rise to the same symptom 7. In some cases, endometrial cancers may reach an advanced stage before signs and symptoms can be noticed. Although the tumor marker CA125 may assist in the detection of EC, its concentration is more likely to be raised in type II or advanced stage cancers than earlier-stage cancers and a normal value does not exclude more advanced tumors 8. According to the SEER (Surveillance, Epidemiology, and End Results) database, the 5-year survival rate of the patients who have distant metastasis slumps to 17% 1. Therefore, there is an urgent need to discover potential early diagnostic and prognostic candidates for the clinicians to refer to when adopting appropriate treatments.

Cell Division Cycle Associated (CDCA) family is composed of eight members, that is, NUF2 (alias: CDCA1), CDCA2/3/4/5, CBX2 (alias: CDCA6), CDCA7/8, which play different roles in cell cycle. NUF2 is a component of the essential kinetochore-associated NDC80 complex, which is required for chromosome segregation and spindle checkpoint activity 9-11. CDCA2 is reported to be involved in nuclear envelope reformation and regulation of the DNA damage response 12. CDCA3 is found to serve as a trigger for the entry into mitosis and mediates the destruction of mitosis-inhibitory kinase wee1 13-15. CDCA4 regulates E2F-dependent transcriptional activation and cell proliferation and is involved in spindle organization from prometaphase 16. CDCA5 functions as a regulator of sister chromatid cohesion in mitosis stabilizing cohesin complex association with chromatin 17. CBX2 takes part in maintaining the transcriptionally repressive state of many genes throughout development via chromatin remodeling and modification of histones 18. CDCA7 participates in MYC-mediated cell transformation and apoptosis 19. CDCA8 is required for chromatin-induced microtubule stabilization and spindle formation 20,21. Any dysregulation in the process of cell division may lead to malignancy 22,23.

Our team previously discovered that CDCA8 acted as hub gene in the tumorigenesis of EC 24. To date, no research has focused on the clinical relevance of any Cell Division Cycle Associated (CDCA) family member in endometrial carcinoma. Therefore, our study was focused on identifying the potential CDCA members with diagnostic and prognostic promise by comprehensive and systematic analysis based on large volume of databases. The relationship between transcriptional expression data of CDCAs and clinical parameters, genetic alterations, biological functional and pathway enrichment analysis were also analyzed to advance our knowledge of the effect of CDCAs on the tumorigenesis and progression of EC.

## Methods

### TCGA UCEC data sources

The gene expression data (575 cases, Workflow Type: HTSeqCounts) and clinical information of the patients were downloaded from The Cancer Genome Atlas (TCGA) official website for the Uterine Corpus Endometrial Carcinoma projects (UCEC). The DESeq2 25 R package was applied to acquire the normalized UCEC RNA-sequencing profile. In the final TCGA-UCEC cohort, 543 patients with intact overall survival (OS), relapse-free survival (RFS) data and complete RNAseq data were enrolled in the present study, of which 23 paracancerous tissues taken from those patients were matched with their corresponding cancerous samples. Intact demographic and clinical parameters were collected and showed in Table S1.

### GEPIA

The online database Gene Expression Profiling Interactive Analysis (GEPIA) is an interactive web that includes 9736 tumors and 8587 normal samples from TCGA and the GTEx projects 26. Differential expression levels of CDCA family were explored by this database and the difference was calculated by Students' t test.

### Survival analysis

TCGA UCEC cohort was divided into two groups based on the best cutpoint gene expression (that corresponds to the most significant relation with survival probability) detected by Survminer R package. The influence of the expression of each CDCA member on the overall survival (OS) and relapse-free survival (RFS) of EC patients was evaluated by Kaplan-Meier method and log-rank test.

### cBioportal data extraction

The cBioPortal 27 for Cancer Genomics provides comprehensive analyses of complex tumor genomics and clinical profiles from The Cancer Genome Atlas (TCGA). We used this tool to analyze genomic alterations (e.g. amplifications, deep deletions, and mutations) of CDCAs in UCEC as a whole and in its various histological subgroups. Co-expressed genes with each CDCA family member were also downloaded from cBioportal. ClusterProfiler R package 28 was applied to perform GO (gene ontology) functional annotation and KEGG (Kyoto Encyclopedia of Gene and Genome) pathway enrichment analysis on the co-expressed genes of CDCA family.

### GeneMANIA

GeneMANIA 29 provides a flexible web interface to generate a list of genes with similar functions with the queried gene and constructs an interactive functional-association network to colorfully illustrate their relationships. In this study, it was adopted to construct a PPI network for CDCA family based on physical interactions, co-expression, pathway and genetic interaction, as well as to evaluate their biological functions.

### NetworkAnalyst

NetworkAnalyst 30 is an online visual analytics platform specialized in transcriptome profiling, network analysis, and meta-analysis for gene expression data. It aimed to address the key need for interpreting gene expression data within the context of protein-protein interaction (PPI) networks, including cell-type or tissue specific PPI networks, gene regulatory networks, gene co-expression networks. In our study, we used this web-tool to assess the up-stream targets (including miRNA and transcription factor targets) of all CDCA family members and built a miRNA-TF regulatory network.

### Statistic method

All the analyses were conducted using R (v.3.5.1). The differential expression levels of CDCA family were compared and analyzed by Students' t test. The difference between the cancerous tissues and their paired paracancerous ones was analyzed by Wilcoxon test. The relationship between clinical pathologic features and the expression level of each CDCA family member was analyzed by Students' t test and logistic regression. The influence of the expression level of each CDCA member on the overall survival (OS) and relapse-free survival (RFS) of EC patients was evaluated by Kaplan-Meier method and log-rank test. The correlation among CDCA family members and the correlation of co-expressed genes with CDCAs were assessed by Spearman test. P < 0.05 indicated statistically significant differences.

## Results

### Aberrant overexpression of CDCA family in patients with EC

**Figure 1:**
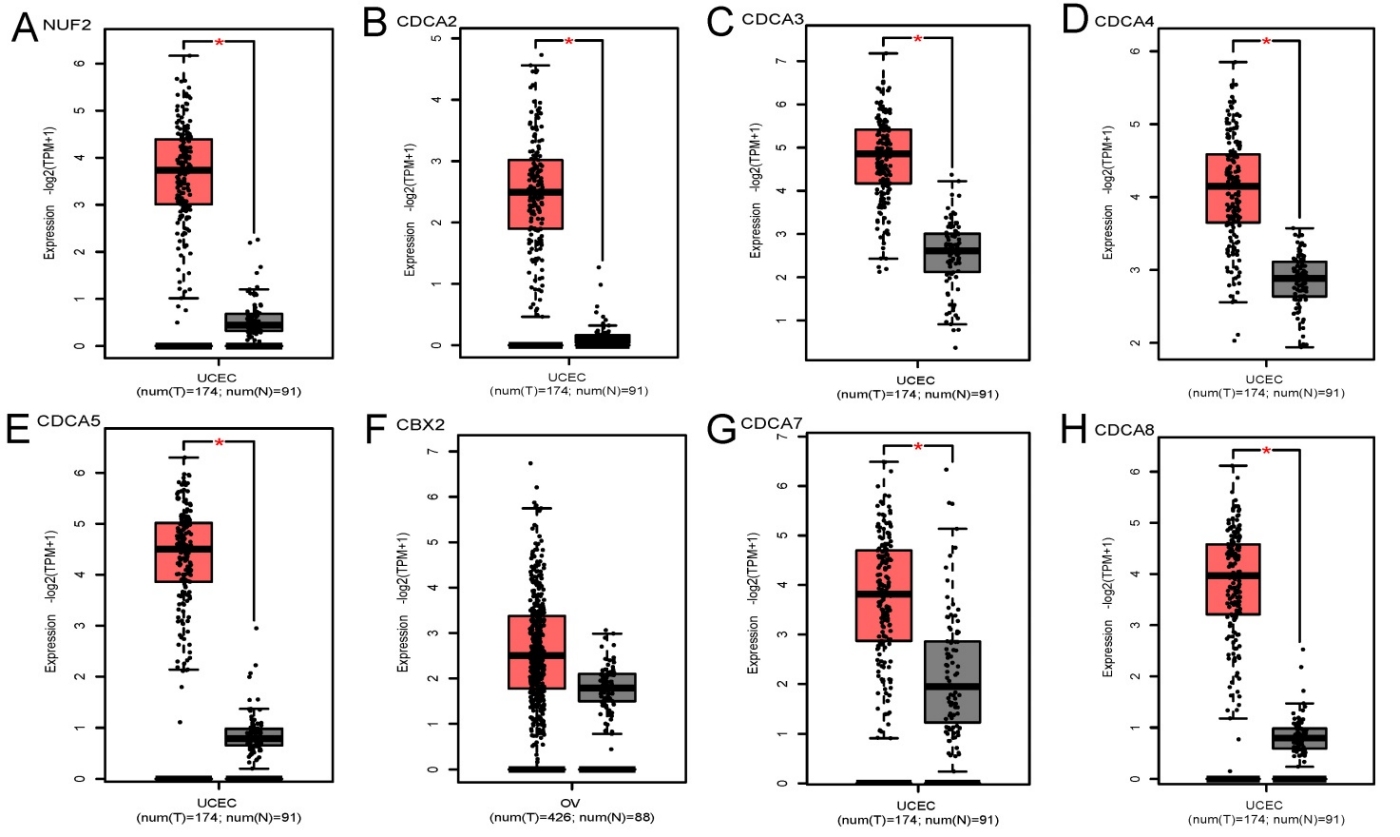
Transcriptional expression levels of CDCA family in EC and normal endometrial tissues (GEPIA database). (A)NUF2, (B)CDCA2, (C)CDCA3 (D)CDCA4, (E)CDCA5, (F)CBX2, (G)CDCA7, (H)CDCA8. Red box represents tumor, grey box normal. Y-axis units are -Log2 (TPM + 1). *P < 0.01.

**Figure 2:**
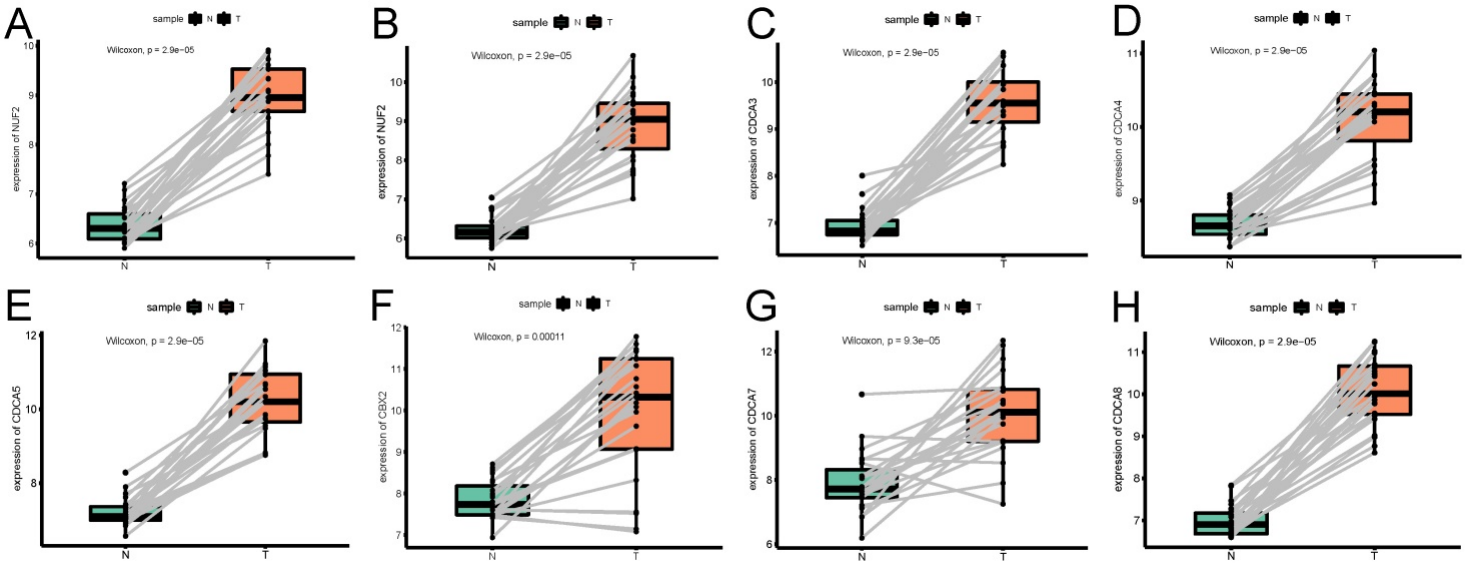
The expression profile of each CDCA member in paired endometrial cancerous samples and the corresponding adjacent normal ones. (A)NUF2, (B)CDCA2, (C)CDCA3 (D)CDCA4, (E)CDCA5, (F)CBX2, (G)CDCA7, (H)CDCA8.

GEPIA database contained 174 cancerous endometrial tissues and 91 normal endometrium samples. As Figure 1 demonstrates, NUF2, CDCA2/3/4/5/7/8 were significantly overexpressed in EC tissues than normal tissues (p<0.001). Also, we investigated the expression difference simply in normalized TCGA UCEC cohort (containing 543 EC samples and 23 paracancerous samples). In contrast with what we found in GEPIA, all the CDCA members were significantly elevated in EC (Fig S1) (p<0.001). To shore up the evidence, EC samples were matched with their adjacent normal ones (Figure 2), which was consistent with the former conclusion.

### The relationship between the transcriptional expression level of each CDCA family member and clinical parameters

**Figure 3:**
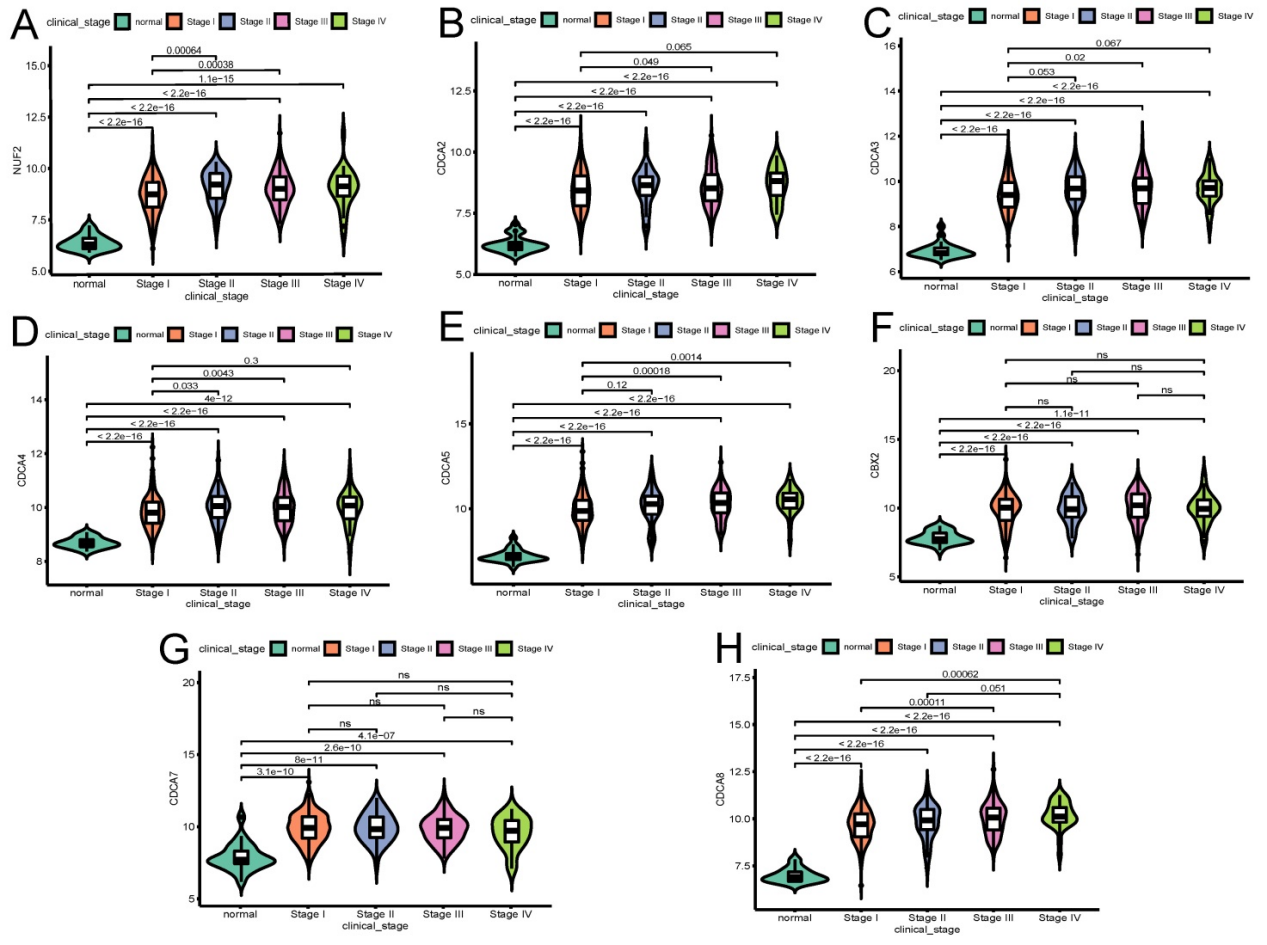
The relationship between the transcriptional expression of each CDCA family member and FIGO stages(A)NUF2, (B)CDCA2, (C)CDCA3 (D)CDCA4, (E)CDCA5, (F)CBX2, (G)CDCA7, (H)CDCA8. ns: not significant.

**Figure 5:**
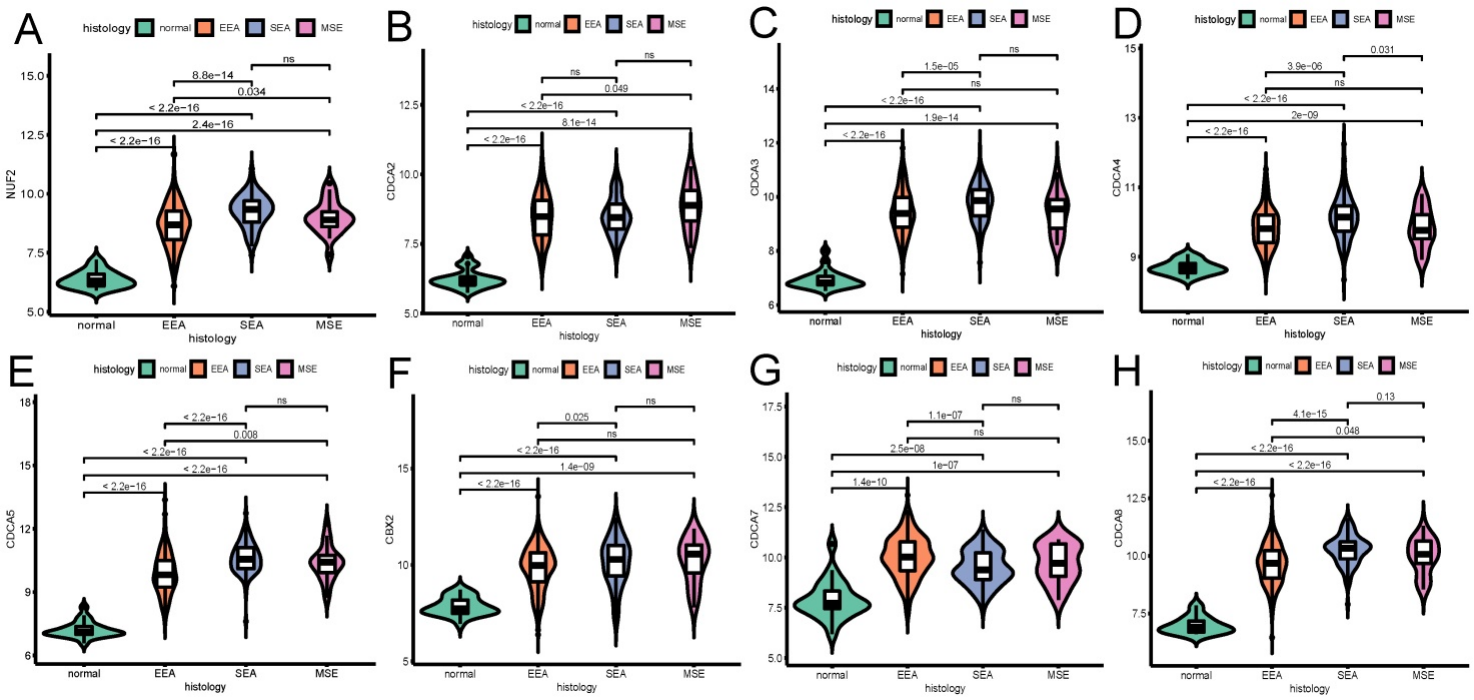
The relationship between the transcriptional expression of each CDCA family member and histologic subtype (A) NUF2, (B) CDCA2, (C) CDCA3 (D) CDCA4, (E) CDCA5, (F) CBX2, (G) CDCA7, (H) CDCA8. ns: not significant. EEA: endometrioid endometrial adenocarcinoma. SEA: serous endometrial adenocarcinoma. MSE: mixed serous and endometrioid adenocarcinoma.

The influence of the transcriptional expression level of each CDCA member on traditional clinical parameters was investigated. As Figure 3 shows, the overexpression of NUF2 (stage II vs I and stage III vs I, both p<0.001), CDCA4 (stage II vs I,p=0.033; stage III vs I, p=0.0043), CDCA5 (stage III vs I,p<0.001; stage IV vs I, p=0.0014) and CDCA8 (stage III vs I and stage IV vs I, p<0.001) significantly matched more advanced FIGO stages. CDCA2 and CDCA3 were found significantly elevated only in stage III vs stage I (p=0.049 and p=0.02, respectively). As to histologic grade, overexpression of all the CDCAs except CDCA7 was significantly associated with poor differentiation (all p<0.001). In terms of histologic subgroup (Figure 5), NUF2, CDCA3/4/5/8 were significantly overexpressed in serous endometroid adenocarcinoma (SEA) than endometroid endometrial adenocarcinoma (EEA) and mixed serous and endometrioid adenocarcinoma (MSE) (all p<0.05), except that overexpressed CDCA7 was significantly associated with EEA.

Univariate logistic regression revealed that high expression of NUF2, CDCA2/3/4/5/8 were significantly associated with multiple poor clinical parameters (Table 1) and served as hazardous genes (OR>1), whereas CDCA7 seemed to be protective (OR<1).

### The prognostic predictive potential of CDCAs

**Figure 6:**
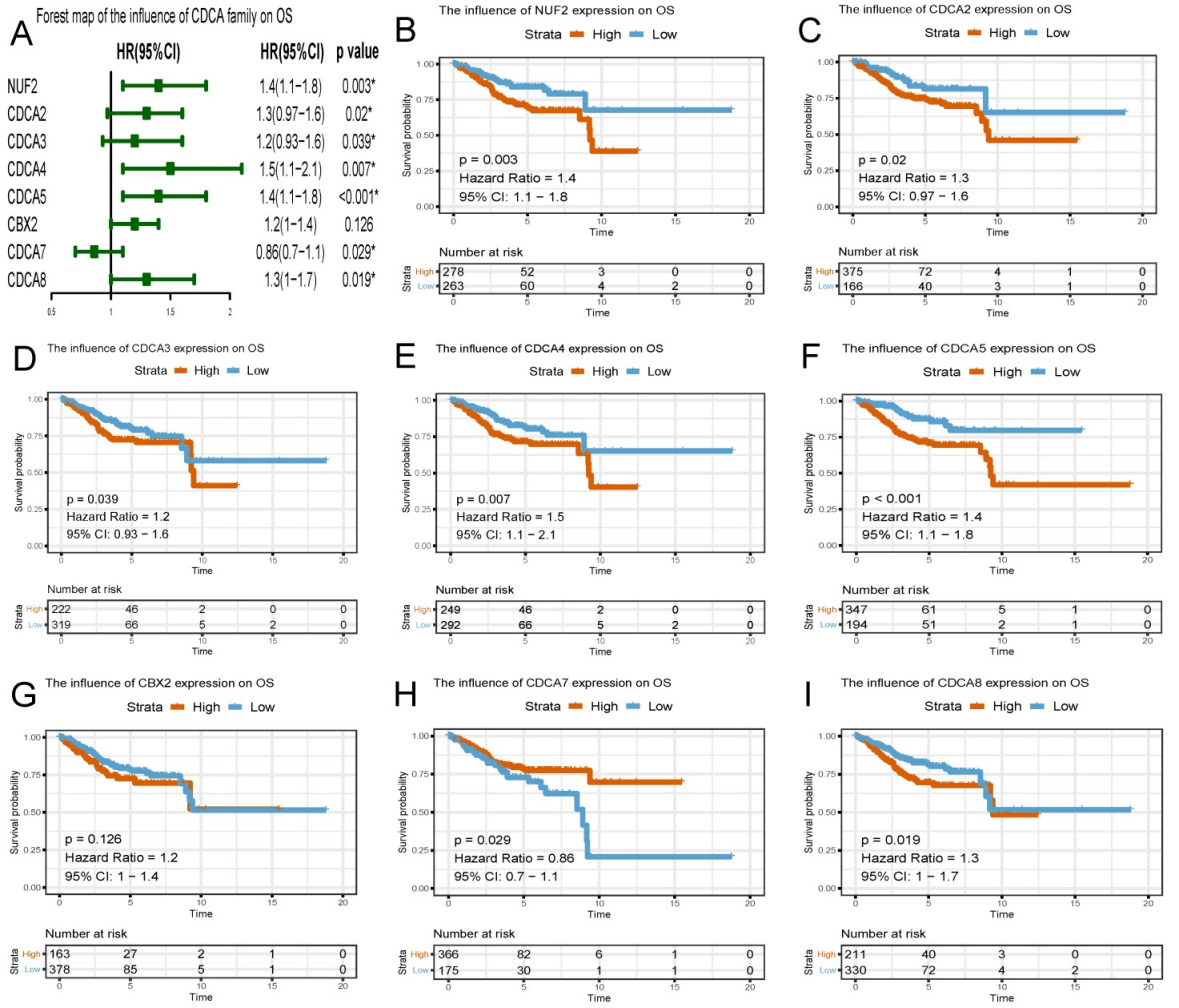
Survival plots of CDCA family and forest map on overall survival (OS) analysis (A~I) Red lines represented higher expression of the gene, blue ones lower expression. X axis means survival time (year). Y axis means survival probability.

To evaluate the effect of differentially expressed CDCAs on the progression of EC, we assessed their correlation with survival outcome. Overall survival curves were presented in Figure 6. The patients with high expression of NUF2 (HR=1.4, 95% CI=1.1-1.8, p=0.003), CDCA2 (HR=1.3, 95% CI=0.97-1.6, p=0.02), CDCA3 (HR=1.2, 95% CI=0.93-1.6, p=0.039), CDCA4 (HR=1.5, 95% CI=1.1-2.1, p=0.007), CDCA5 (HR=1.4, 95% CI=1.1-1.8, p<0.001), CDCA8 (HR=1.3, 95% CI=1.0-1.7, p=0.019) had worse prognosis than those with low expression.

**Figure 7:**
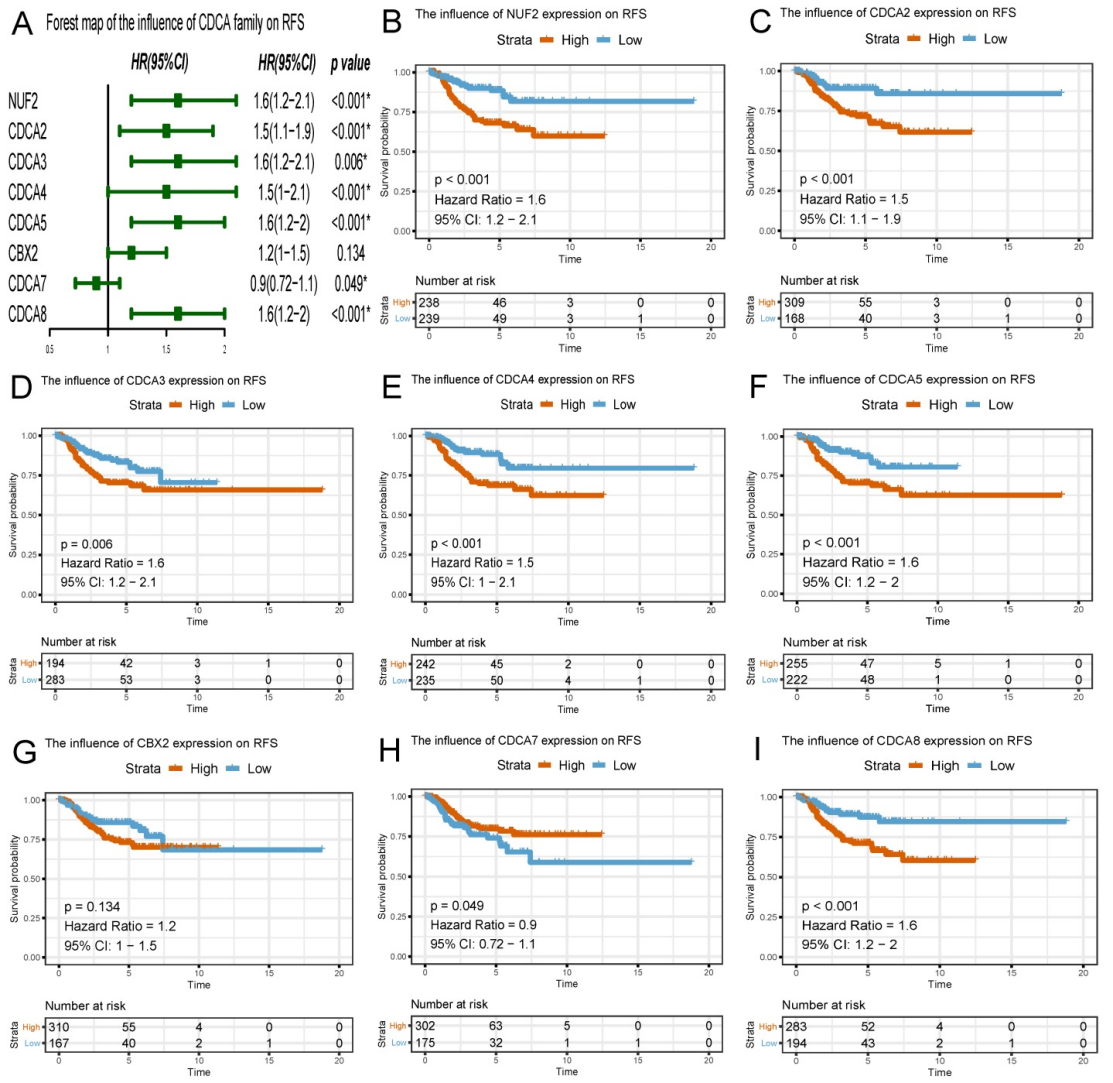
Survival plots of CDCA family and forest map on relapse-free survival (RFS) analysis (A~I) Red lines represented higher expression of the gene, blue ones lower expression. X axis means survival time (year). Y axis means survival probability.

Similar to the results in OS analyses, overexpressed NUF2 (HR=1.6, 95%CI=1.2-2.1, p<0.001), CDCA2 (HR=1.5, 95% CI=1.1-1.9, p<0.001), CDCA3 (HR=1.6, 95% CI=1.2-2.1, p=0.006), CDCA4 (HR=1.5, 95% CI=1.0-2.1, p<0.001), CDCA5 (HR=1.6, 95% CI=1.2-2.0, p<0.001), CDCA8 (HR=1.6, 95% CI=1.2-2, p<0.001) were also significantly related with poor survival outcome in RFS analyses (Figure 7). By contrast, overexpressed CDCA7 was significantly with better prognosis in OS (HR=0.86, 95% CI=0.7-1.1, p=0.029) and RFS (HR=0.9, 95% CI=0.72-1.1, p=0.049) analyses. Nevertheless, CBX2 expression did not exert significantly prognostic influence on EC patients (neither OS nor RFS).

### Genetic alterations underlying abnormal expression of CDCA family in EC patients

**Figure 8:**
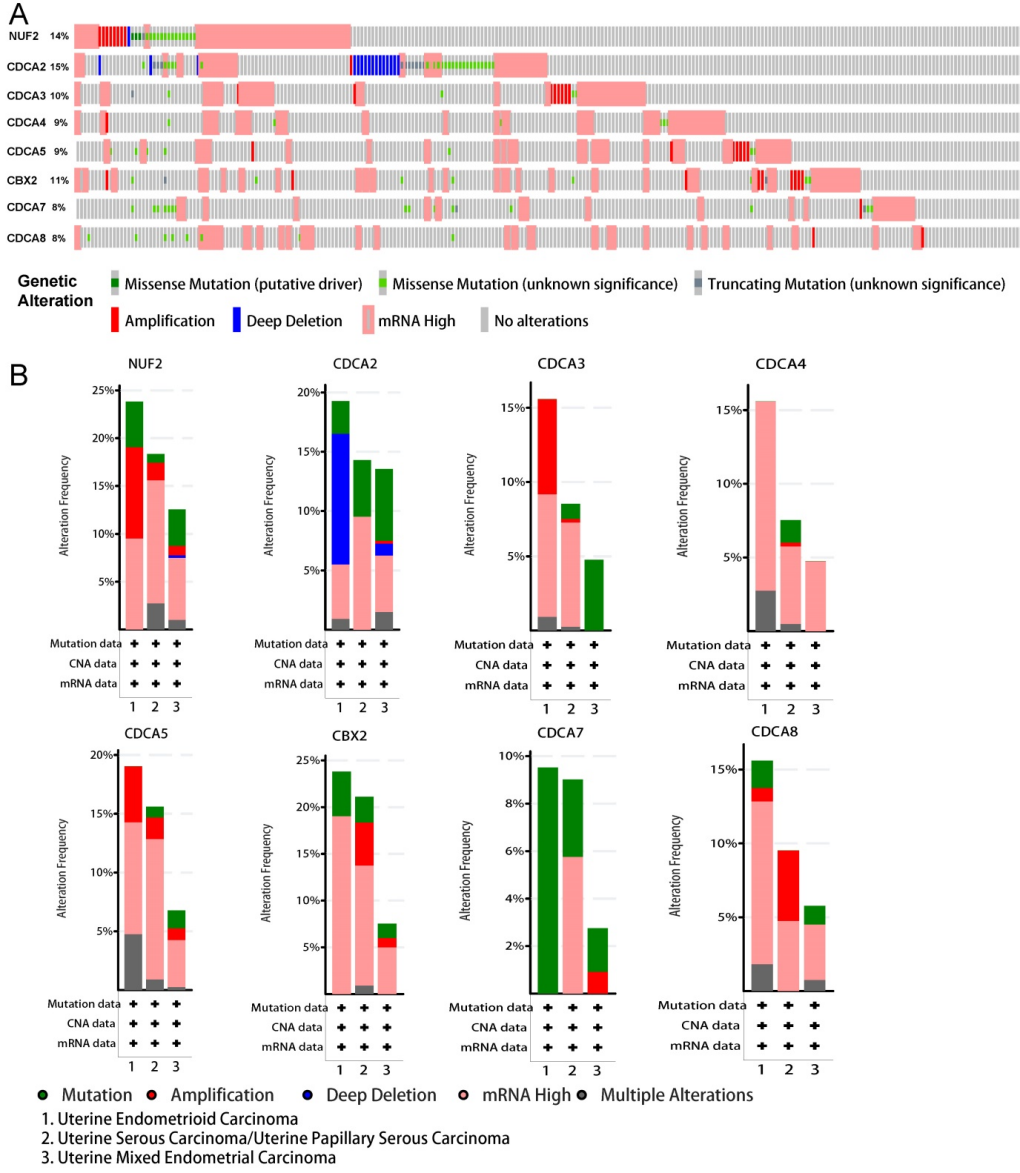
Genomic alterations behind abnormal expression of CDCA family in EC (cBioPortal). (A) OncoPrint visual summary of variations on query of CDCA family members (B**)** Analyses of genetic variations of every CDCA family member in various subgroup.

To gain an in-depth insight into the molecular mechanisms of differential expression of CDCAs, genetic alterations were analyzed in EC patients as a whole and in various histologic subtypes. As Figure 8A shows, CDCA2 possessed the highest probability of the alterations (15%), followed by NUF2 (14%). Generally, high mRNA expression accounted for the most. When these alterations were grouped by different histologic subtypes (i.e. Uterine Endometrioid Carcinoma, Uterine Serous Carcinoma/Uterine Papillary Serous Carcinoma and Uterine Mixed Endometrial Carcinoma), we observed distinct patterns of genetic variations (Figure 8B). In uterine endometrioid carcinoma, the prevalent high mRNA expression of almost all CDCAs except CDCA7, the amplification of NUF2, CDCA3/5, the deep deletion of CDCA2 and the missense mutation of CDCA7 presented the most common altered genetic events. As to uterine serous carcinoma/uterine papillary serous carcinoma, the most common alterations were the high mRNA expression of all CDCA members, the amplification of CDCA5, CBX2 and CDCA8 and the missense mutation of CDCA2/7. When it comes to uterine mixed endometrial carcinoma, another distinctive pattern emerged and the most were the high mRNA expression of almost all CDCA members except CDCA3/7, and the missense mutation of NUF2, CDCA2/3/5, CBX2, CDCA7/8.

### Correlations among CDCA family members and protein-protein interaction (PPI) network

**Figure 9:**
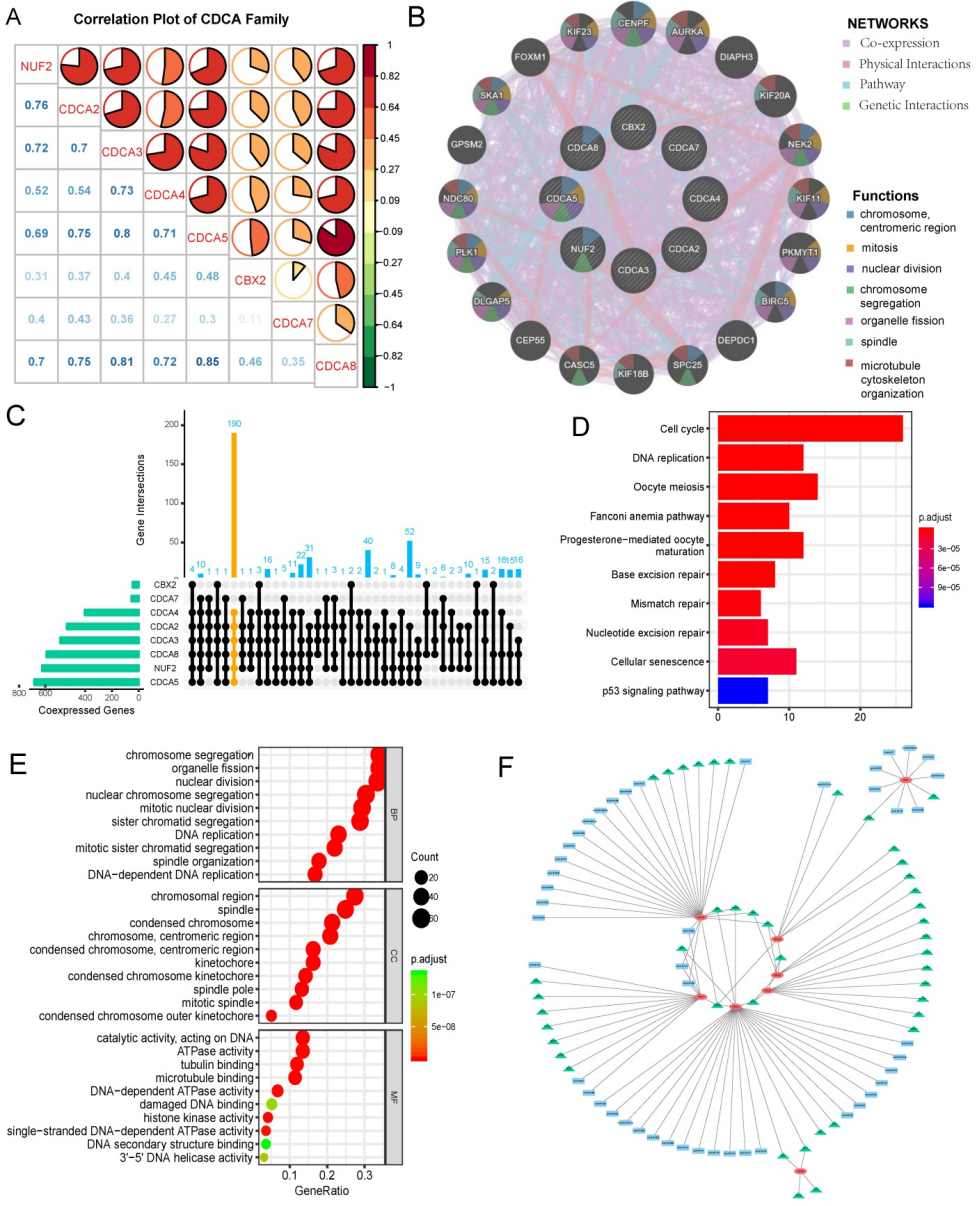
Correlations among CDCA family members, protein-protein interaction(PPI) network construction, assessment of up-stream targets and down-stream signaling pathways of CDCAs in EC (A) Correlation plot among CDCA family members. The color of the pie chart represents the correlation of the expression of two genes(red: positively correlated, green: negatively correlated). The area of the colored pie is proportional to the strength of mutual correlation. The blue numbers in the lower part represent the specific correlation coefficient(by Spearman test). (B)PPI network and functional analysis from GENEMANIA (C)UpSetR plot illustrating the numbers of co-expressed genes shared by different sets of CDCA family members, with yellow highlighting the co-expressed genes with NUF2,CDCA2/3/4/5/8 (D) Enriched KEGG pathways of the shared co-expressed genes with NUF2, CDCA2/3/4/5/8 (E)Top 10 enriched GO terms of co-expressed genes with NUF2, CDCA2/3/4/5/8. BP: biological process; MF: molecular function; CC: cellular component. (F) The regulatory network of miRNA and transcription factor targets of CDCAs. The green triangle represents possible transcription targets. The blue rectangle represents possible miRNA targets.

Spearman test was used to determine the correlation power among CDCA family members in EC. The coefficient exceeding 0.60 was thought to indicate strong correlation. As shown in Figure 9A, strong positive correlation existed between NUF2 with CDCA2/3/58; CDCA2 with CDCA3/5/8; CDCA3 with CDCA4/5/8; CDCA4 with CDCA5/8; CDCA5 with CDCA8 (all p=0.000). Moreover, we constructed a PPI network (Figure 9B) of CDCAs by GENEMANIA to explore the potential interactions among them and it was built on the grounds of the following characteristics: co-expression, physical interactions, pathway and genetic interactions. CENPF, AURKA, DIAPH3, KIF20A, NEK2, KIF11, PKMYT1, BIRC5, DEPDC1, SPC25, KIF18B, CASC5, CEP55, DLGAP5, PLK1, NDC80, GPSM2, SKA1, FOXM1 and KIF23 were the neighboring genes predicted to interact with CDCA family. The functional analysis by this tool revealed CDCAs along with their potentially interacting genes took effect in the following process: chromosome, mitosis, nuclear division, chromosome segregation, organelle fission, spindle and microtubule cytoskeleton organization (Table S2). CDCA5 was assumed to play multifaceted roles in the aforementioned process.

### Assessment of up-stream targets and down-stream signaling pathways of CDCAs in EC

Co-expressed genes with every CDCA family member were defined as those with Spearman correlation coefficient greater than 0.40 and p value less than 0.001. UpSetR plot was applied to illustrate the number of co-expressed genes shared by different sets of CDCA family members (Figure 9C). Given the predictive values of overexpressed NUF2, CDCA2/3/4/5/8 for worse prognosis of EC, their common co-expressed genes were extracted to perform pathway and functional annotation enrichment analyses. KEGG (Kyoto Encyclopedia of Gene and Genome) analysis results (Figure 9D) demonstrated those co-expressed genes were mainly enriched in cell cycle, DNA replication, oocyte meiosis, Fanconi anemia pathway, progesterone-mediated oocyte maturation, base excision repair, mismatch repair, nucleotide excision repair, cellular senescence and p53 signaling pathway (p<0.001). GO (gene ontology) analysis results (Figure 9E) were divided into three parts: biological process (BP), molecular function (MF) and cellular component (CC). Those co-expressed genes were mainly enriched in chromosome segregation organelle fission(BP), nuclear division(BP), nuclear chromosome segregation (BP), mitotic nuclear division (BP), sister chromatid segregation (BP) chromosomal region (CC), spindle (CC), condensed chromosome (CC), chromosome (CC), condensed chromosome (CC), catalytic activity (MF), ATPase activity (MF), tubulin binding (MF), microtubule binding (MF) and DNA-dependent ATPase activity (MF) (p<0.001). We also explored possible transcription factor targets and miRNA targets of CDCAs using the Networkanalyst database. As Fig 9F and Table 2 shows, TFAP2A (Transcription Factor AP-2 Alpha) was associated with the regulation of CDCA4, CDCA5, CBX2, CDCA7. E2F1 was predicted to be key transcription factor for CDCA3/5/7. SP1 was the key transcription factor for CDCA2/3/5. NFYA was the key transcription factor for CDCA2, CDCA3, CDCA4. In terms of miRNA targets, hsa-miR-30b, hsa-miR-124, hsa-miR-30a were the main key miRNA targets for CBX2, CDCA7.

## Discussion

Endometrial carcinoma (EC) is the most common cancer of female reproductive organs. Abnormal uterine bleeding is the most frequent symptom of EC, but many other disorders give rise to the same symptom 7. Although the tumor marker CA125 may assist in the detection of EC, it still has limitations in the diagnosis of earlier-stage cancers and a normal value does not exclude more advanced tumors 8. Therefore, there is an urgent need to discover potential early diagnostic and prognostic biomarkers for the clinicians to refer to when adopting appropriate measures.

Cell Division Cycle Associated (CDCA) family is made up of eight members, that is, NUF2 (alias: CDCA1), CDCA2/3/4/5, CBX2 (alias: CDCA6), CDCA7/8. Each member plays different or synergistic roles in the process of cell cycle. Cumulative studies have demonstrated that any dysregulation in the process of cell division may lead to malignancy 22,23. Previous studies have reported the indispensable role of CDCAs in the tumorigenesis of clear cell renal cell carcinoma 31 and lung carcinoma 32. Our team previously discovered that CDCA8 could act as hub gene in the tumorigenesis of EC and was associated with poor prognosis 24. However, the role and the clinical relevance of whole Cell Division Cycle Associated (CDCA) family in EC remain elusive.

For the first time, our study investigated the transcriptional expression profile of CDCAs in TCGA UCEC mRNA seq data alone and in combination with GTEx projects. The preliminary results showed NUF2, CDCA2, CDCA3, CDCA4, CDCA5, CDCA7, CDCA8 were significantly overexpressed in EC tissues than normal tissues. Nevertheless, in TCGA UCEC cohort alone (543 EC samples plus 23 paracancerous samples), all the CDCA members were significantly elevated in EC (p<0.001), which was further corroborated in paired EC and adjacent normal samples.

The most important prognostic features in EC are the FIGO stage, histological type, and differentiation grade; most are independent of each other 33-35. Consequently, the present study also explored the expression level of CDCAs with clinical characteristics and found that the expression of NUF2 (stage III vs I, p<0.001), CDCA2/3/4 (stage III vs I: p=0.049, p=0.02, p=0.0043, respectively), CDCA5 (stage IV vs I, p=0.0014), CDCA8 (stage IV vs I, p<0.001) significantly increased as the FIGO stages advanced. Of note, all CDCAs expression levels in any FIGO stage (especially stage I) of EC patients were significantly higher than that in normal tissues (p<0.001), signifying their potential as early detective biomarkers. Besides, the overexpression of all the CDCAs except CDCA7 (associated with well differentiation) was significantly associated with poor differentiation (p<0.05). Since serous endometroid adenocarcinoma (SEA) possesses more aggressive nature and often relates with poor prognosis, our research showed overexpression of NUF2, CDCA3/4/5, CBX2 and CDCA8 were significantly associated with SEA (p<0.05). In addition, univariate logistic regression revealed that high expression of NUF2, CDCA2/3/4/5/8 were significantly associated with multiple poor clinical parameters and served as hazardous genes (OR>1), whereas CDCA7 seemed to be protective (OR<1). These results imply that highly expressed NUF2, CDCA2/3/4/5/8 may exert oncogenic effects on EC patients.

Survival analysis using Kaplan Meier method also provided convincing evidence, as is seen that the patients with high expression of NUF2 (OS: HR=1.4, RFS: HR=1.6), CDCA2 (OS: HR=1.3, RFS: HR=1.5), CDCA3 (OS: HR=1.2, RFS: HR=1.6), CDCA4 (OS: HR=1.5, RFS: HR=1.5), CDCA5 (OS: HR=1.4, RFS: HR=1.6), CDCA8 (OS: HR=1.3, RFS: HR=1.6) had worse prognosis than those with low expression (p<0.05). Therefore, it is assumed that NUF2, CDCA2/3/4/5/8 could serve as promising predictive candidates, which may offer more evidence for the prediction of survival outcome in EC patients with accuracy.

Interestingly, although CDCA7 was found to be significantly highly expressed in EC than normal tissues (p<0.001), its high overexpression was significantly related with some better prognostic characteristics (poor vs well differentiation: OR=0.554 (0.332-0.925), p=0.024; SEA vs EEA: OR=0.349 (0.227-0.535), p=0.000) and also with better OS (HR=0.86, 95% CI=0.7-1.1, p=0.029) and RFS (HR=0.9, 95% CI=0.72-1.1, p=0.049). Gill et al. 19 reported CDCA7 could be phosphorylated by AKT and sequestrated to the cytoplasm. Induction of CDCA7 expression in the presence of MYC sensitized cells to apoptosis upon serum withdrawal, whereas CDCA7 knockdown reduced MYC-dependent apoptosis. This may explain the role that CDCA7 plays in EC, and further validation by experiments is required, though.

The tumorigenesis and progression of EC is complicated and multi-faceted, and genetic alteration plays an important role in this process, so we explored the molecular characteristics of CDCAs in EC. Our study revealed that frequent genetic alterations prevailed in CDCA family and each member exhibited diverse patterns of variations in different subtypes of EC. In general, elevated mRNA expression accounted for the most.

In order to illustrate the interactions of neighboring genes and CDCAs, we constructed a PPI network. GENEMANIA results showed CENPF, AURKA, DIAPH3, KIF20A, NEK2, KIF11, PKMYT1, BIRC5, DEPDC1, SPC25, KIF18B, CASC5, CEP55, DLGAP5, PLK1, NDC80, GPSM2, SKA1, FOXM1 and KIF23 were the neighboring genes predicted to interact with CDCAs. We also found a low to high correlation among CDCAs, suggesting that NUF2 and CDCA2/3/4/5/8 might play synergistic role in the onset and progression of EC. No literature or experiments have been carried out on their interaction and the relevant regulatory mechanisms in EC, which points new direction to the current research.

To identify the CDCAs-involved biological pathways in EC, co-expressed genes with CDCAs with predictive capability for worse survival outcome (i.e. NUF2, CDCA2/3/4/5/8) were extracted to conduct GO and KEGG enrichment analyses. GO functional enrichment analysis showed those genes were mainly enriched in chromosome segregation organelle fission (BP), chromosomal region (CC), catalytic activity (MF) (p<0.001). KEGG pathway enrichment analysis demonstrated that the following pathways were mainly enriched: cell cycle, DNA replication, oocyte meiosis, Fanconi anemia pathway, progesterone-mediated oocyte maturation, base excision repair, mismatch repair, nucleotide excision repair, cellular senescence and p53 signaling pathway (p<0.001). As is well known, endometrial carcinoma originates from the aberrant growth of the endometrium. The enrichment evidence strongly consolidates the assumption that CDCAs can affect the proliferation and apoptosis of endometrial cancer cells through the aforementioned pathways and thus regulate the onset and progression of EC.

Combined with KEGG enrichment analysis results we have stated above, all the CDCAs were significantly overexpressed in EEA (type I EC) than normal endometrium and NUF2, CDCA3/4/5/8 were highly-expressed in SEA (belonging to type II EC) than EEA (type I EC) and normal ones, it is reasonable to assume estrogen(or its receptor) might play role in the overexpression of CDCAs in type I EC and p53 signaling pathway could take effect in the high expression of NUF2, CDCA3/4/5/8 in type II EC. However, cytologic experiments are required to assess the effect of estrogen (or its receptor) and p53 signaling pathways in the overexpression of CDCAs in EC.

In summary, our study clarified the clinical relevance and the potential biological functions of CDCA family in EC. Survival probability and the relationship between their transcriptional expression level and clinical parameters were analyzed in TCGA UCEC cohort. NUF2 and CDCA2/3/4/5/8 showed great promise in early diagnosis and prognostic prediction for EC patients. Genomic alterations and biological functions were analyzed to explore the potential mechanisms of the aberrant expression of CDCAs in the oncogenesis of EC. Although this study is preliminary bioinformatic results, it offers new direction for the future research. Our team will address the biological behavior and molecular mechanism of CDCAs in EC by cytologic experiments, which will greatly advance our understanding and provide better implications for treating patients with efficacy.

## Supplementary Material

Supplementary figures and tables.

## Figures and Tables

**Figure 4:**
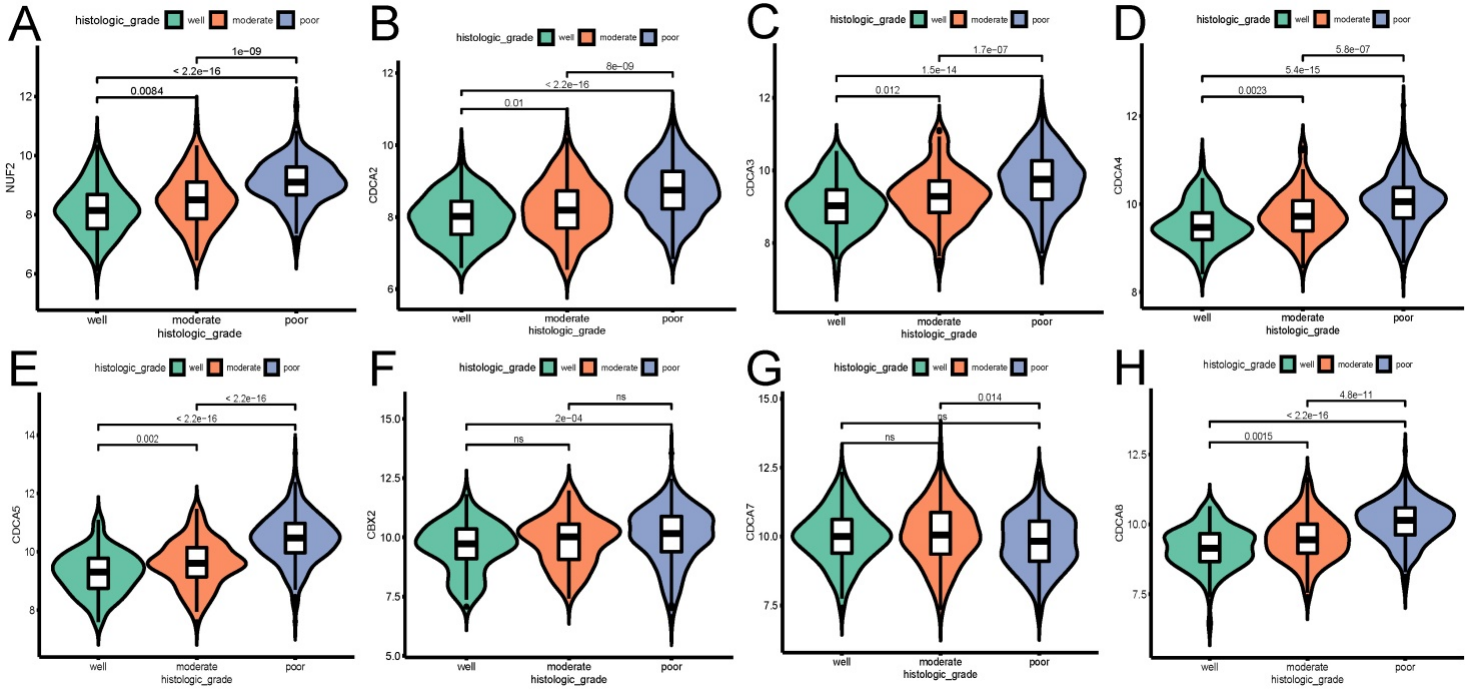
The relationship between the transcriptional expression of each CDCA family member and histologic grade (A) NUF2, (B) CDCA2, (C) CDCA3 (D) CDCA4, (E) CDCA5, (F) CBX2, (G) CDCA7, (H) CDCA8. ns: not significant.

**Table 1:** CDCA family member expression associated with clinical parameters (binary logistic regression)

		Clinical parameters							
		NUF2		CDCA2		CDCA3		CDCA4	
		Odds ratio	p-value	Odds ratio	p-value	Odds ratio	p-value	Odds ratio	p-value
Stage	II vs I	2.374 (1.277-4.414)	0.006*	1.669 (0.841-3.310)	0.143	1.732 (0.958-3.132)	0.069	2.342 (1.282-4.279)	0.006*
	III vs I	1.699 (1.120-2.577)	0.013*	1.416 (0.896-2.237)	0.136	1.802 (1.188-2.732)	0.006*	1.720 (1.136-2.602)	0.010*
	IV vs I	1.943 (0.891-4.237)	0.095	1.968 (0.779-4.969)	0.152	1.930 (0.901-4.134)	0.09	2.473 (1.132-5.399)	0.023*
Histologic grade	Moderate vs well	1.727 (0.941-3.171)	0.078	1.696 (0.989-2.908)	0.055	1.785 (0.910-3.502)	0.092	2.079 (1.109-3.897)	0.022*
	Poor vs well	7.038 (4.151-11.932)	0.000*	4.436 (2.745-7.169)	0.000*	6.054 (3.395-10.795)	0.000*	5.927 (3.428-10.245)	0.000*
	Poor vs moderate	4.075 (2.613-6.355)	0.000*	2.616 (1.661-4.121)	0.000*	3.391 (2.134-5.390)	0.000*	2.851 (1.838-4.423)	0.000*
Histologic subtype	SEA vs EEA	3.696 (2.323-5.883)	0.000*	1.407 (0.879-2.251)	0.154	2.432 (1.593-3.713)	0.000*	2.533 (1.647-3.895)	0.000*
		**CDCA5**		**CBX2**		**CDCA7**		**CDCA8**	
		**Odds ratio**	**p-value**	**Odds ratio**	**p-value**	**Odds ratio**	**p-value**	**Odds ratio**	**p-value**
Stage	II vs I	2.023 (1.055-3.882)	0.034*	1.363 (0.726-2.558)	0.336	1.009 (0.535-1.902)	0.978	1.876 (1.034-3.401)	0.038*
	III vs I	2.399 (1.509-3.814)	0.000*	1.721 (1.115-2.656)	0.014*	0.934 (0.602-1.448)	0.759	2.325 (1.528-3.537)	0.000*
	IV vs I	6.635 (1.970-22.346)	0.000*	1.038 (0.444-2.427)	0.931	0.755 (0.344-1.653)	0.482	2.989 (1.379-6.478)	0.006*
Histologic grade	Moderate vs well	1.815 (1.023-3.220)	0.042*	1.418 (0.711-2.825)	0.321	0.931 (0.503-1.723)	0.82	2.602 (1.152-5.879)	0.021*
	Poor vs well	13.806 (8.100-23.530)	0.000*	3.080 (1.723-5.506)	0.000*	0.554 (0.332-0.925)	0.024*	11.974 (5.831-24.589)	0.000*
	Poor vs moderate	7.607 (4.757-12.166)	0.000*	2.173 (1.333-3.543)	0.002*	0.595 (0.373-0.949)	0.029*	4.601 (2.814-7.524)	0.000*
Histologic subtype	SEA vs EEA	18.167 (7.259-45.467)	0.000*	1.783 (1.154-2.755)	0.009*	0.349 (0.227-0.535)	0.000*	5.003 (3.196-7.830)	0.000*

**Table 2:** Key regulated transcription factors and miRNA targets of CDCAs (Networkanalyst)

Key TF	Description	Regulated gene
MXI1	MAX Interactor 1	CDCD4, CDCA7
TFAP2C	Transcription Factor AP-2 Gamma	CDCA4, CDCA7
E2F2	E2F Transcription Factor 2	CDCA3, CDCA7
E2F1	E2F Transcription Factor 1	CDCA3, CDCA5, CDCA7
SP1	Sp1 Transcription Factor	CDCA2, CDCA3, CDCA5
NFYA	Nuclear Transcription Factor Y Subunit Alpha	CDCA2, CDCA3, CDCA4
TFAP2A	Transcription Factor AP-2 Alpha	CDCA4, CDCA5, CBX2, CDCA7
MAX	MYC Associated Factor X	CDCA4, CDCA8
EBF1	EBF Transcription Factor 1	CDCA4, CDCA8
GABPA	GA Binding Protein Transcription Factor Subunit Alpha	NUF2, CDCA5
EGR1	Early Growth Response 1	CDCA4, CBX2
Key miRNA	Description	Regulated gene
hsa-miR-30b	-	CBX2, CDCA7
hsa-miR-124	-	CBX2, CDCA7
hsa-miR-30a	-	CBX2, CDCA7

## Abbreviations

EC: Endometrial carcinoma
CDCA: Cell Division Cycle Associated
NUF2: Component Of NDC80 Kinetochore Complex
CBX2: chromobox 2
TCGA: The Cancer Genome Atlas
GO: gene ontology
KEGG: Kyoto Encyclopedia of Gene and Genome
UCEC: Uterine corpus endometroid carcinoma
OS: overall survival
RPS: relapse-free survival
PPI: protein-protein interaction
FIGO: International Federation of Gynecology and Obstetrics
UCEC: uterine corpus endometrial carcinoma
BP: biological process
MF: molecular function
CC: cellular component
OR: odds ratio
HR: hazard ratio
