# Supplementary material for: Systematic Analysis of the Clinical Relevance of Cell Division Cycle Associated Family in Endometrial Carcinoma
Source: J Cancer. 2020 Jul 20;11(19):5588–600. doi: 10.7150/jca.46324 (PMC7477460; doi:10.7150/jca.46324)
Supplement: Supplementary file 1 — Supplementary figures and tables. [file jcav11p5588s1.pdf]

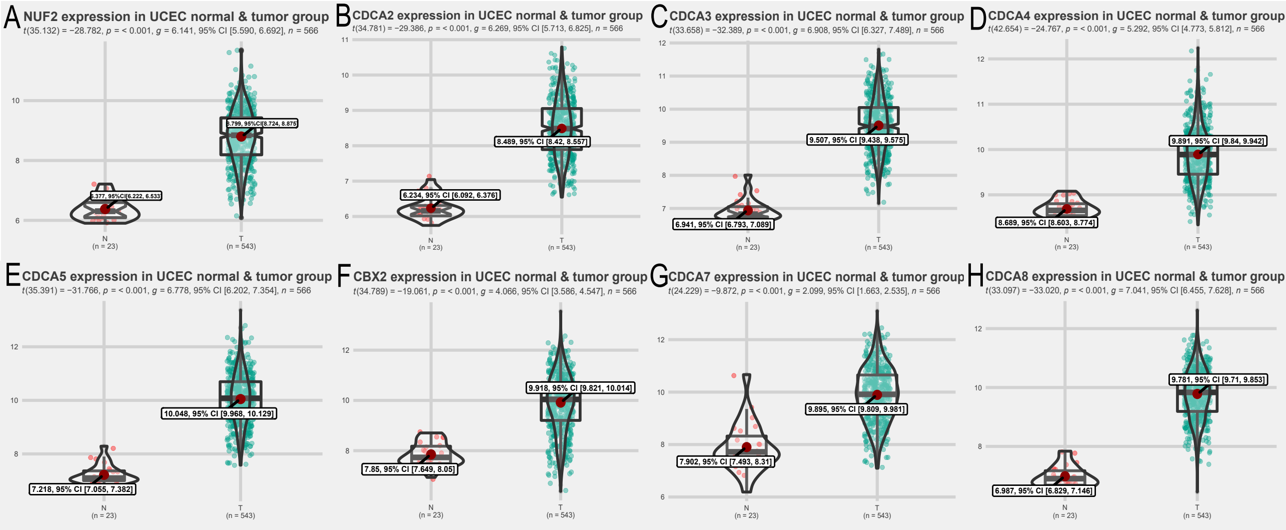

A NUF2 expression in UCEC normal & tumor group
t(35.132) = −28.782, p = < 0.001, g = 6.141, 95% CI [5.590, 6.692], n = 566
N (n = 23)
T (n = 543)
B CDCA2 expression in UCEC normal & tumor group
t(34.781) = −29.386, p = < 0.001, g = 6.269, 95% CI [5.713, 6.825], n = 566
6.234, 95% CI [6.092, 6.376]
8.489, 95% CI [8.42, 8.557]
N (n = 23)
T (n = 543)
C CDCA3 expression in UCEC normal & tumor group
t(33.658) = −32.389, p = < 0.001, g = 6.908, 95% CI [6.327, 7.489], n = 566
6.941, 95% CI [6.793, 7.089]
9.507, 95% CI [9.438, 9.575]
N (n = 23)
T (n = 543)
D CDCA4 expression in UCEC normal & tumor group
t(42.654) = −24.767, p = < 0.001, g = 5.292, 95% CI [4.773, 5.812], n = 566
8.689, 95% CI [8.603, 8.774]
9.891, 95% CI [9.84, 9.942]
N (n = 23)
T (n = 543)
E CDCA5 expression in UCEC normal & tumor group
t(35.391) = −31.766, p = < 0.001, g = 6.778, 95% CI [6.202, 7.354], n = 566
7.218, 95% CI [7.055, 7.382]
10.048, 95% CI [9.968, 10.129]
N (n = 23)
T (n = 543)
F CBX2 expression in UCEC normal & tumor group
t(34.789) = −19.061, p = < 0.001, g = 4.066, 95% CI [3.586, 4.547], n = 566
7.85, 95% CI [7.649, 8.05]
9.918, 95% CI [9.821, 10.014]
N (n = 23)
T (n = 543)
G CDCA7 expression in UCEC normal & tumor group
t(24.229) = −9.872, p = < 0.001, g = 2.099, 95% CI [1.663, 2.535], n = 566
7.902, 95% CI [7.493, 8.31]
9.895, 95% CI [9.809, 9.981]
N (n = 23)
T (n = 543)
H CDCA8 expression in UCEC normal & tumor group
t(33.097) = −33.020, p = < 0.001, g = 7.041, 95% CI [6.455, 7.628], n = 566
6.987, 95% CI [6.829, 7.146]
9.781, 95% CI [9.71, 9.853]
N (n = 23)
T (n = 543)

**Table S1. clinical parameters of patients with endometrial carcinoma in TCGA cohort**

| **Clinical parameters** | | **Total (543)** | **%** |
|---|---|---|---|
| **Age at diagnosis** | | 63(33-90) | |
| FIGO cancer staging system | I | 339 | 62.43% |
| | II | 51 | 9.39% |
| | III | 124 | 22.83% |
| | IV | 29 | 5.34% |
| Histological grade | well | 98 | 18.05% |
| | moderate | 120 | 22.09% |
| | poor | 325 | 59.86% |
| Histologic subtype | EEA | 407 | 74.95% |
| | MSE | 22 | 4.05% |
| | SEA | 114 | 21.00% |

FIGO: International Federation of Gynecology & Obstetrics; EEA:endometroid endometrial adenocarcinoma; MSE: mix serous and endometrioid carcinoma; SEA: serous endometrial adenocarcinoma

Table S2

| Function | FDR | Genes in network | Genes in genome |
|---|---|---|---|
| mitosis | 1.32E-13 | 12 | 180 |
| chromosome, centromeric region | 7.34E-13 | 10 | 98 |
| nuclear division | 3.18E-12 | 12 | 256 |
| chromosome segregation | 5.80E-12 | 10 | 128 |
| organelle fission | 5.87E-12 | 12 | 281 |
| spindle | 1.04E-11 | 10 | 141 |
| microtubule cytoskeleton organization | 1.42E-11 | 11 | 220 |
| protein-DNA complex | 2.49E-11 | 10 | 158 |
| kinetochore | 1.79E-10 | 8 | 74 |
| condensed chromosome kinetochore | 6.93E-10 | 6 | 22 |
| regulation of cell division | 9.28E-10 | 9 | 153 |
| condensed chromosome, centromeric | 1.78E-09 | 6 | 26 |
| spindle microtubule | 2.50E-08 | 6 | 40 |
| midbody | 2.50E-08 | 7 | 81 |
| condensed chromosome | 3.53E-08 | 7 | 86 |
| regulation of mitosis | 6.74E-08 | 7 | 95 |
| regulation of nuclear division | 9.82E-08 | 7 | 101 |
| spindle organization | 6.46E-07 | 6 | 70 |
| condensed chromosome outer kinetochore | 1.13E-06 | 4 | 11 |
| G2/M transition of mitotic cell cycle | 1.42E-06 | 7 | 152 |
| cell cycle G2/M phase transition | 1.42E-06 | 7 | 152 |
| establishment of chromosome localization | 1.61E-05 | 4 | 21 |
| spindle pole | 1.61E-05 | 5 | 59 |
| chromosome localization | 1.61E-05 | 4 | 21 |
| microtubule | 4.51E-05 | 6 | 149 |
| regulation of mitotic cell cycle | 9.94E-05 | 7 | 290 |
| mitotic spindle organization | 0.000123 | 4 | 35 |
| cell cycle phase | 0.000228 | 4 | 41 |
| spindle checkpoint | 0.000295 | 4 | 44 |
| cytokinesis | 0.000323 | 5 | 112 |
| mitotic sister chromatid segregation | 0.000428 | 4 | 49 |
| sister chromatid segregation | 0.000616 | 4 | 54 |
| establishment of organelle localization | 0.00077 | 5 | 136 |
| attachment of spindle microtubules to kinetochore | 0.001439 | 3 | 19 |
| organelle localization | 0.001598 | 5 | 160 |
| M phase | 0.001598 | 3 | 20 |
| positive regulation of cell cycle process | 0.002507 | 5 | 177 |
| microtubule binding | 0.00253 | 4 | 80 |
| protein localization to chromosome | 0.002608 | 3 | 24 |
| positive regulation of cell cycle | 0.003208 | 4 | 86 |
| positive regulation of mitosis | 0.004831 | 3 | 30 |
| microtubule anchoring | 0.004831 | 3 | 30 |
| positive regulation of nuclear division | 0.006323 | 3 | 33 |
| organelle assembly | 0.006904 | 5 | 226 |
| spindle assembly | 0.007234 | 3 | 35 |
| spindle assembly checkpoint | 0.008385 | 3 | 37 |
| metaphase/anaphase transition of mitotic cell cycle | 0.010405 | 3 | 40 |
| metaphase/anaphase transition of cell | 0.010982 | 3 | 41 |
| microtubule polymerization or depolymerization | 0.011343 | 3 | 42 |
| tubulin binding | 0.011343 | 4 | 125 |
| regulation of mitotic metaphase/anaphase transition | 0.013706 | 3 | 45 |

| | | | |
|---|---|---|---|
| regulation of metaphase/anaphase transition of cell cycle | 0.014367 | 3 | 46 |
| protein kinase binding | 0.014943 | 5 | 276 |
| centrosome organization | 0.033945 | 3 | 62 |
| regulation of microtubule cytoskeleton organization | 0.039453 | 3 | 66 |
| microtubule organizing center organization | 0.039453 | 3 | 66 |
| spindle assembly involved in mitosis | 0.052329 | 2 | 13 |
| regulation of microtubule-based process | 0.070012 | 3 | 81 |
| cell cycle checkpoint | 0.070114 | 4 | 209 |
| maintenance of protein location in cell | 0.073818 | 3 | 84 |
| regulation of centrosome cycle | 0.073818 | 2 | 16 |
| metaphase plate congression | 0.073818 | 2 | 16 |
| maintenance of location in cell | 0.090903 | 3 | 91 |
| mitotic cytokinesis | 0.090989 | 2 | 18 |
| maintenance of protein location | 0.090989 | 3 | 92 |
